# PSMA-PET-derived distance features as biomarkers for predicting outcomes in primary prostate cancer post-radical prostatectomy

**DOI:** 10.1186/s40644-025-00907-8

**Published:** 2025-07-22

**Authors:** Ruohua Chen, Ye Li, Dong Liang, Jianjun Liu, Tao Sun

**Affiliations:** 1 Department of Nuclear Medicine, Shanghai Jiao Tong University School of Medicine Affiliated Renji Hospital, 160 Pujian Road, Shanghai, China; 2 Shenzhen Institutes of Advanced Technology, Chinese Academy of Sciences, 1068 Xueyuan Avenue, Shenzhen, Guangdong China

**Keywords:** PSMA-PET, Prostate cancer, Biochemical recurrence, Outcome prediction

## Abstract

**Objectives:**

This study aims to assess the predictive capability of PSMA-PET imaging for disease outcomes in primary prostate cancer post-radical prostatectomy. In addition to conventional lesion uptake measures, the evaluation includes the distance of lesion to the prostate to enhance risk stratification and outcome prediction.

**Methods:**

A cohort of 190 men diagnosed with primary prostate cancer and undergoing prostatectomy were initially screened, resulting in 103 patients meeting the selection criteria. Imaging parameters, including lesion SUVmax, primary metabolic tumor volume (PMTV), maximum distance from the lesion to the prostate (Dmax), and total distances from the lesion to the prostate (Dtotal), were extracted from 68Ga-PSMA-11 PET images. Findings were dichotomized based on primary lesion uptake, the tumor volume size, Dmax distance, and the presence of metastatic disease. Postoperative biochemical recurrence-free survival (BCRFS) was analyzed using Kaplan–Meier survival plots and Log-rank tests. Furthermore, univariate and multivariate Cox regression analyses were performed to evaluate the association of PET parameters with survival outcomes.

**Results:**

Clinical and histopathological characteristics were summarized, including age, weight, height, metastasis status, baseline PSA, biopsy Gleason score, pt stage, margin status, and lymph node status. After a median follow-up of 20 months, 66 events occurred, with the estimated 3-year BCRFS being 46%. Increased PSMA intensity (SUVmax > 17.06) was associated with less favorable BCRFS (log-rank *p* = 0.017). Increased primary metabolic tumor volume (PMTV > 41.59 cm^3^) was also linked to less favorable BCRFS (log-rank *p* = 0.003). Dmax and Dtotal greater than 9.69 cm and 11.95 cm were identified as negative prognostic factors for BCRFS (log-rank *p* < 0.001 and *p* = 0.002, respectively). Based on PMTV and Dmax, patients were stratified into low-, intermediate-, and high-risk groups, with 3-year BCRFS rates of 57%, 31%, and 8%, respectively. Univariate Cox regression analysis revealed significant associations between BCRFS and factors such as baseline PSA (HR: 1.69, 95% CI 1.02–2.79, *p* = 0.042), SUVmax (HR: 1.56, 95% CI 1.04–1.91, *p* = 0.018), PMTV (HR: 2.05, 95% CI 1.26–3.34, *p* = 0.004), Dmax (HR: 2.24, 95% CI 1.37–3.65, *p* = 0.001), and Dtotal (HR: 2.11, 95% CI 1.29–3.45, *p* = 0.003). Multivariable Cox regression analysis identified the best model with PMTV (HR: 2.57, *p* = 0.004) and Dmax (HR: 1.98, *p* = 0.009) as independent predictors for biochemical recurrence (C-index = 0.68).

**Conclusion:**

The lesion distance to prostate was defined and assessed in conjunction with conventional PET parameters to facilitate preoperative risk stratification in primary prostate cancer following radical prostatectomy. The findings contribute to improved outcome prediction and emphasize the potential of PSMA-PET imaging in enhancing management strategies for prostate cancer patients.

**Clinical relevance:**

There is a critical need for non-invasive biomarkers that can predict treatment outcomes for patients with primary prostate cancer. Our study introduces the concept of using distance metrics, specifically the lesion distance to prostate in baseline PSMA-PET scans, to improve the prediction of biochemical recurrence following prostatectomy. These distance metrics consider the spatial distribution of lesions, offering a novel approach to assessing tumor spread and its implications for patient outcomes.

## Introduction

Approximately 30–40% of prostate cancer patients experience failure of primary treatment and subsequently require additional disease management [1]. Preoperative serum prostate-specific antigen (PSA) levels [2] and biopsy Gleason score (GS) [3] are established risk factors commonly used to predict biochemical recurrence (BCR) following radical prostatectomy.

Imaging biomarkers are gathering increasing interest in enhancing the accuracy of biochemical recurrence prediction. Multiparametric MRI, used as a prognostic nomogram, can predict clinical outcomes in patients with primary prostate cancer post-prostatectomy [4, 5]. In FDG-PET imaging, a prostate SUVmax ≥ 4.6 is associated with a twofold risk of biochemical recurrence one year post-surgery [6]. Additionally, positive results from 11C-choline PET/CT have been shown to predict prostate cancer-specific survival in patients following prostatectomy [7].

The increased utilization of PSMA-PET scans in primary staging has been suggested to correlate with decreased BCR-free survival (BCRFS) [8, 9]. Presurgical PSMA-PET can serve as a robust prognostic biomarker [10, 11]. For instance, the intensity of PSMA in the primary tumor independently predicts BCRFS post-radical prostatectomy [12–14]. In PSMA-PET imaging, metabolic tumor volume (MTV) quantifies the total volume of PSMA-avid tumor regions throughout the body, offering a comprehensive assessment of tumor burden compared to SUVmax [15, 16]. Patients with higher tumor burden face elevated risks of treatment failure and shorter survival durations following prostatectomy [17, 18]. However, MTV does not account for the spatial distribution of lesions within the body.

The objective of this retrospective study is to investigate the potential of distance parameters derived from 68Ga-PSMA-11 PET as non-invasive biomarkers for predicting biochemical recurrence post-radical prostatectomy. Specifically, the distance from the lesion to the prostate center was defined to account for the spatial distribution of lesions. Alongside primary lesion SUVmax and MTV, the study assesses the ability of these parameters to enhance preoperative risk stratification.

**Fig. 1:**
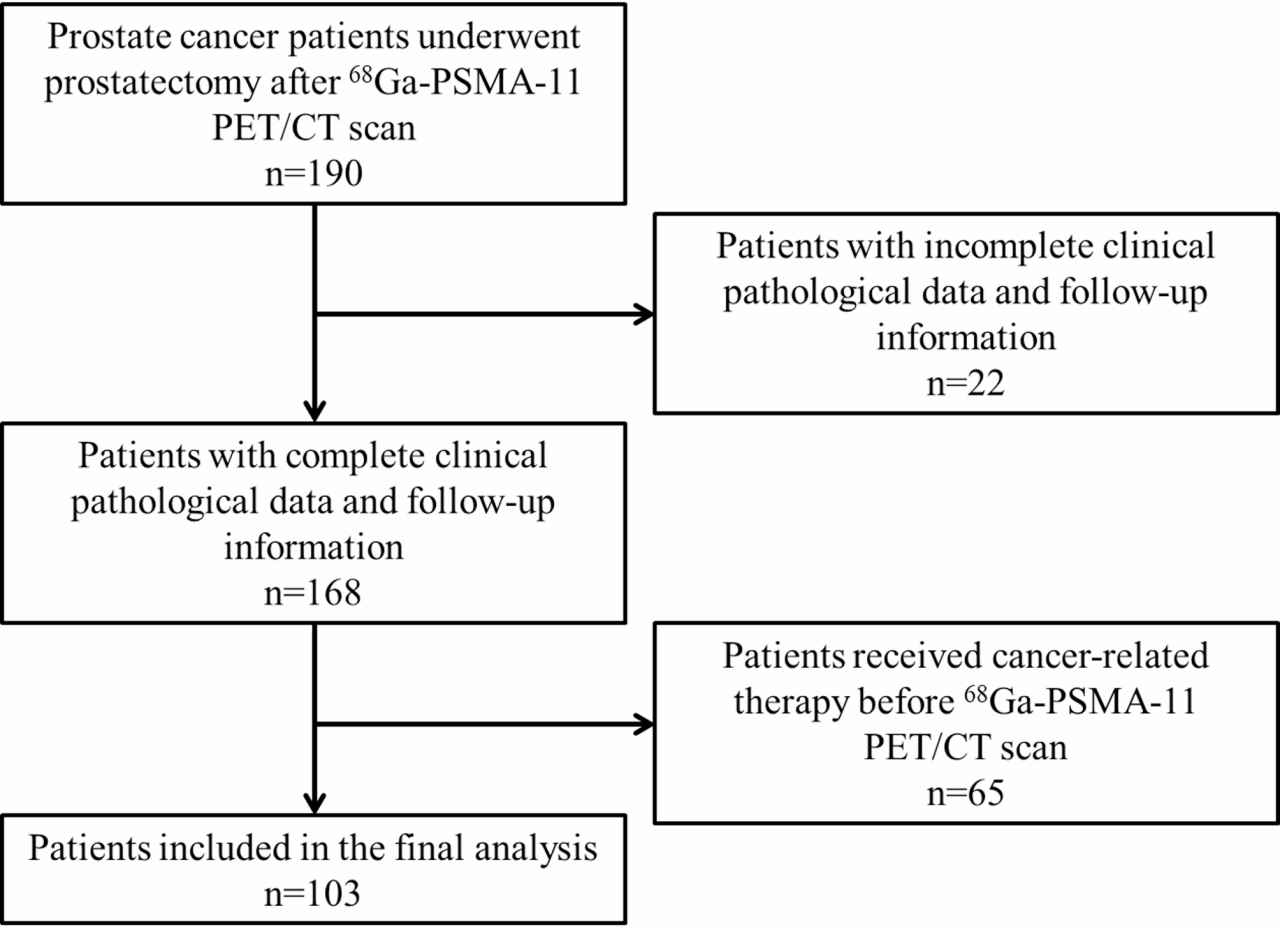
Flow chart of patient selection

## Materials and methods

### Patient selection

This retrospective study was conducted following approval from the Ethics Committee of Shanghai Renji Hospital (IRB 2018 − 104). Informed consent was obtained from all individual participants. We performed a comprehensive screening of the hospital’s database to identify patients who underwent 68Ga-PSMA-11 PET imaging within three months prior to radical prostatectomy from June 2018 and August 2021. The criteria for patient selection are outlined in Fig. 1. Data collection for each subject included age, weight, height, baseline serum PSA levels, and biopsy Gleason scores obtained before surgery. Pathological and metastasis status were assessed according to the Prostate Cancer Molecular Imaging Standardized Evaluation recommendations [19].

### Imaging protocol

The synthesis of 68Ga-PSMA-11 was performed according to the method described in [20]. Patients received an intravenous injection of 68Ga-PSMA-11 with a median administered dose of 135.4 MBq (interquartile range [IQR], 121.1–146.9 MBq). PET acquisition commenced at a median of 60 min post-injection (IQR, 54–68 min). Imaging was distributed over three PET/CT scanner models, specifically a Biograph mCT (Siemens Medical Solutions, Erlangen, Germany), a uMI 780, and a uExplorer PET/CT scanner (United Imaging Healthcare, Shanghai, China) at a single institution. The duration of each scan was 3 min on the Biograph mCT and uMI 780, and 5 min on the uExplorer scanner.

### Image processing

PET/CT scans were reviewed by a nuclear medicine physician with over 20 years of experience, using MIM software (version 7, MIM Software Inc.). PET findings were subsequently communicated to the referring surgeon. SUVs for both primary and metastatic lesions were normalized to body weight and injection dosage, with the maximum SUV of the primary lesion referred to as SUVmax.

For tumor burden, tumor regions were segmented using a 40% SUVmax threshold, a process manually performed by a nuclear medicine physician with 8 years of experience. The primary metabolic tumor volume (PMTV) was defined as the metabolic volume of the primary lesion. Subjects with more than two lesions were classified as having metastasis. The prostate was automatically delineated in the CT images from the PET/CT scan using TotalSegmentator [21]. Spatial distribution features of the tumor were computed based on the distances between each lesion and the prostate. Each lesion was defined by its center as defined by the centroid of the lesion, and distances were calculated using the Euclidean distance between the lesion center and the geometric center of the prostate. The sum of all these distances was termed Dtotal, and the maximum distance was termed Dmax.

### Outcome measure

All included patients underwent radical prostatectomy with standard pelvic lymph node dissection. Biochemical persistence and recurrence were determined by an exhaustive review of all accessible medical records, including PSA levels and clinical documentation. The primary outcome measured was the time to BCR, calculated from the date of surgery. BCR was defined as an increase in PSA levels to over 0.2 ng/mL, occurring at least six weeks after prostatectomy, signifying cancer recurrence or progression following an initial response to treatment. Post-surgery PSA levels were monitored through regularly scheduled appointments as determined by the attending physician. Additionally, clinical notes were reviewed to assess patient symptoms, any further treatments administered, and signs of disease progression.

### Statistical analysis

Statistical analyses were performed using the Statistics and Machine Learning Toolbox in MATLAB (R2021b, MathWorks, MA, United States). Continuous variables were described using mean and ± standard deviation (SD), as well as the median and interquartile range, representing the 25th (Q1) and 75th (Q3) percentiles. A significance threshold of *p* = 0.05 was used to determine statistical significance.

The 68Ga-PSMA-11 PET results, including primary lesion uptake, primary tumor volume, distance measurements, and the presence of metastatic disease, were dichotomized into categories. The Pearson correlation was applied to evaluate the relationships between continuous parameters. The Kendall tau correlation coefficient was applied to evaluate the relationships between distance measures and discrete pathological parameters (GS). Gaussian distribution assumption was tested with Kolmogorov-Smirnov test.

The predictive capability of PET findings on clinical outcomes was validated through Kaplan–Meier survival plots and log-rank tests utilizing Lifelines v0.29. Associations between outcome and PET findings were investigated using both univariate and multivariate logistic regression analyses. Corresponding odds ratios (OR) and 95% confidence intervals (CI) were computed. Parameters demonstrating significant associations in the univariate analysis were included in the multivariate model, with the concordance index (C-index) reported.

**Table 1:** Patient characteristics including demographic and baseline clinical variables. Values are presented as mean ± standard deviation, with median and interquartile range (IQR) in brackets

Subject characteristics	Mean ± SD [median, interquartile range 1–3]
Age (years old)	69.3 ± 6.6 [69, 65–74]
Weight (kg)	69.4 ± 9.3 [69, 64.5–75]
Height (cm)	169.4 ± 5.5 [170, 165.5–173]
Baseline PSA (ng/ml)	72.2 ± 225.8 [36.7, 15.1–84.5]
Baseline Gleason	7.6 ± 0.95 [7, 7–8]
$\:\le\:6$	4
7	48
8	29
$\:\ge\:9$	22
Follow-up time (month)	19.9 ± 6.94 [18.3, 16.2–24.7]
Biochemical relapse at last visit	
Yes	66
No	37
pT stage	
T1/T2/T3/T4	2/48/43/10
Margin status	
Yes	53
No	49
uncertain	1
Lymph node status	
Yes	65
No	38

**Table 2:** Quantitative imaging features derived from 68Ga-PSMA-11 PET, including suvmax, primary metabolic tumor volume (PMTV), and distance metrics (Dmax, Dtotal)

Characteristics PSMA-PET	Mean ± SD [median, interquartile range 1–3]
SUVmax	28.4 ± 19.9 [23.1, 13.7–39.1]
MTV (cm^3^)	68.9 ± 367.2 [25.7, 14.8–50.9]
Dmax (cm)	25.23 ± 35.58 [9.24, 1.56–24.6]
Dtotal (cm)	40.05 ± 61.14 [11.61, 1.59–48.39]

## Results

### Patient characteristics

A total of 190 men diagnosed with prostate cancer and who underwent prostatectomy were initially considered for inclusion in the study. The specific criteria for subject selection are outlined in Fig. 1. The clinical and histopathological characteristics of the 103 patients who met these criteria are summarized in Table 1. The cohort had a median age of 69 years (IQR 65–74). The median baseline PSA level was 36.7 ng/mL (IQR 15.1–84.5), and 99 subjects (96%) had a biopsy Gleason score of ≥ 3 + 4. Among the cohort, 48 patients were staged as T2, 43 as T3, and 10 as T4, with 53 cases showing capsular invasion and 38 exhibiting lymph node metastases. For 68Ga-PSMA imaging, 40 patients were scanned with the Biograph mCT, 39 patients with the uMI PET/CT 780, and 24 patients with the uExplorer PET/CT, resulting in a diverse assessment across different scanner models.

The quantitative measures derived from the PSMA-PET images are detailed in Table 2. The median SUVmax was 23.1 (IQR 13.7–39.1), while the median PMTV was 25.7 cm³ (IQR 14.8–50.9). PET imaging identified focal uptake outside the prostate in 56 patients (54.4%), revealing a total of 217 metastatic lesions. The median maximum lesion distance to the prostate (Dmax) was 9.24 cm (IQR 1.56–24.6), and the total lesion distance to the prostate (Dtotal) was 11.61 cm (IQR 1.59–48.3).

### Univariable effect analysis

**Fig. 2:**
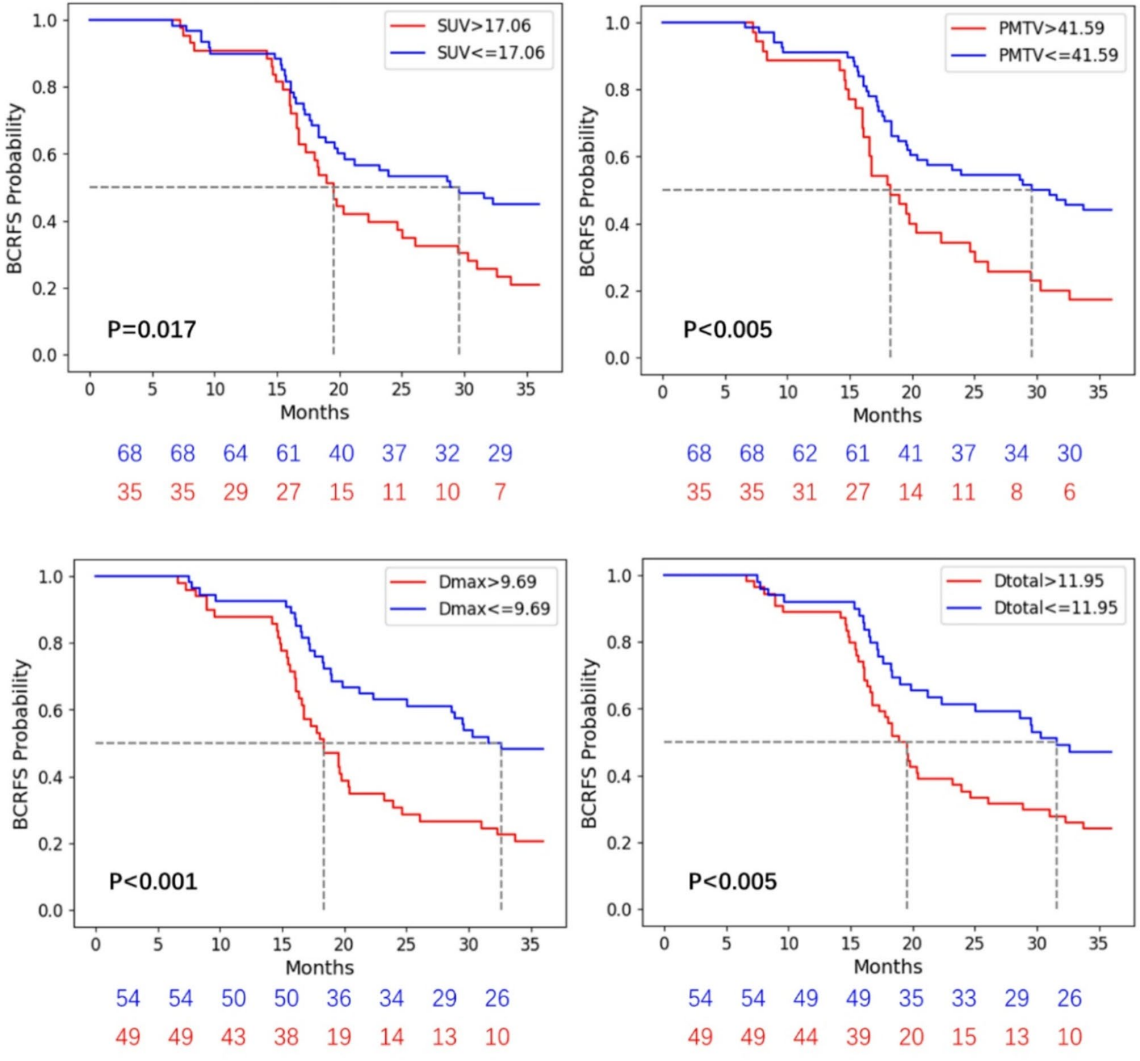
Kaplan Meier estimates of BCRFS with PET features: SUVmax, PMTV, Dmax, and Dtotal. High- and low-risk patient groups can be stratified based on thresholding PET-derived features (*p* < 0.05)

Over a 36-month follow-up period, 66 patients (64%) experienced BCR. An increased PSMA intensity (SUVmax > 17.1) was significantly associated with poorer BCRFS (log-rank *p* = 0.017). Conversely, patients exhibiting low PSMA uptake in the primary lesion were less likely to experience recurrence during the follow-up period (Fig. 2). Additionally, a higher primary metabolic tumor volume (PMTV > 41.59 cm³) was correlated with decreased BCRFS (log-rank *p* = 0.003). Distance metrics even played a more crucial role; a Dmax exceeding 9.69 cm emerged as a negative prognostic indicator for BCRFS (log-rank *p* < 0.001), while a Dtotal greater than 11.95 cm was similarly a negative predictor for BCRFS (log-rank *p* = 0.002). There is a significantly high correlation between Dmax and Dtotal (*r* = 0.94). Both Dmax and Dtotal significantly correlated with PSA and PMTV measures (r **=** 0.28 and 0.49 for Dmax; *r* = 0.42 and 0.28 for Dtotal). No significant correlations were found between Dmax/Dtotal and SUVmax/Gleason score (*p* > 0.05).

Table 3 presents the results from univariate Cox regression analysis, examining preoperative imaging risk factors for BCR. Several factors were significantly associated with BCR-free survival in prostate cancer patients post-prostatectomy: baseline PSA level (hazard ratio [HR]: 1.69, 95% CI 1.02–2.79, *p* = 0.042); image-based features including SUVmax (HR: 1.56, 95% CI 1.04–1.91, *p* = 0.018), PMTV (HR: 1.76, 95% CI 1.09–2.84, *p* = 0.004), Dmax (HR: 2.24, 95% CI 1.37–3.65, *p* = 0.001), and Dtotal (HR: 2.11, 95% CI 1.29–3.45, *p* = 0.003). Other clinical, pathological, or imaging measures are not independent prognostic factors.

**Table 3:** Univariable Cox regression analysis for biochemical recurrence-free survival (BCRFS) in prostate cancer patients post-radical prostatectomy. HR: hazard ratio; CI: confidence interval

Characteristics	HR [95% CI]	*P*-value
Age (> 66.21 y)	0.87 [0.53, 1.44]	0.596
Weight (> 68.43 kg)	1.31 [0.81, 2.13]	0.271
PSA (> 68.38 ng/ml)	1.69 [1.02, 2.79]	**0.042**
Gleason score (> 8.0)	1.61 [0.89, 2.92]	0.112
Pt stage (> T2)	0.67 [0.43, 1.05]	0.081
Margin status (> 0)	0.79 [0.51, 1.12]	0.277
Lymph node status (> 0)	1.15 [0.78, 2.01]	0.069
SUVmax (> 17.06)	1.56 [1.04, 1.91]	**0.018**
PMTV (> 41.59 cm^3^)	2.05 [1.26, 3.34]	**0.004**
Dmax (> 9.69 cm)	2.24 [1.37, 3.65]	**0.001**
Dtotal (> 11.95 cm)	2.11 [1.29, 3.45]	**0.003**

### Combination of distance feature with uptake index

**Fig. 3:**
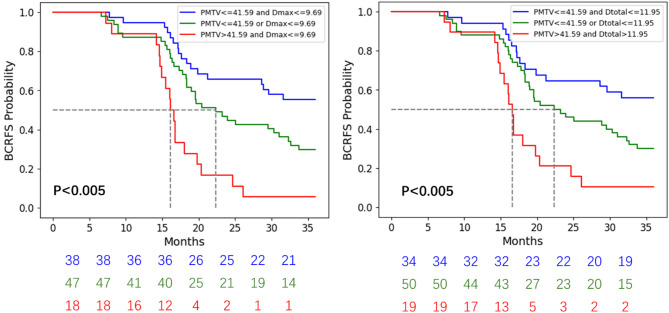
Kaplan Meier estimates of BCRFS when combining PMTV and Dmax (Dtotal) enable the stratification of patients into three risk groups

The stratification of patients at risk was further optimized based on the presence or absence of high Dmax and PMTV values. As depicted in Fig. 3 (left), the 103 subjects were categorized into three distinct subgroups: low-risk Group 1, intermediate-risk Group 2, and high-risk Group 3. Group 1 included patients with PMTV < 41.59 cm³ and Dmax < 9.69 cm (*n* = 24), Group 2 comprised patients with either PMTV < 41.59 cm³ or Dmax ≥ 9.69 cm (*n* = 36), and Group 3 consisted of patients with both PMTV ≥ 41.59 cm³ and Dmax ≥ 9.69 cm (*n* = 24). The log-rank tests revealed significant differences among the groups (*p* = 0.028, *p* < 0.005, *p* < 0.005, respectively). The newly classified groups exhibited 3-year recurrence-free rates of 57%, 29%, and 6%, respectively. Similar results were found when combining Dtotal and PMTV as shown in Fig. 3 (right).

**Fig. 4:**
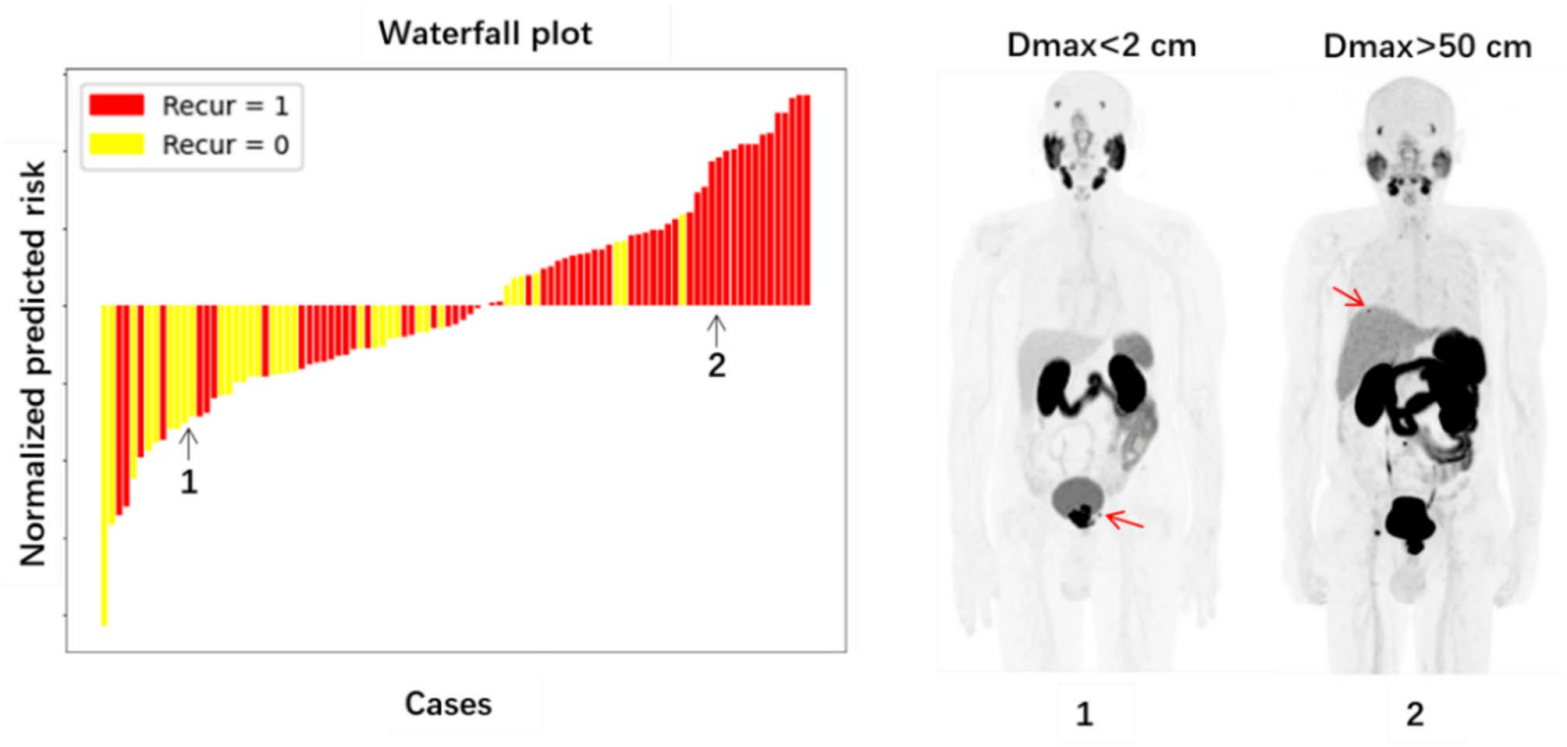
The left is the waterfall plot that shows the predicted risk for the multivariable Cox model. The right demonstrates two cases, one with recurrence and one without. They were accompanied by low (< 9.69 cm) and high (> 9.69 cm) Dmax in PSMA-PET scans, respectively

Table 4 presents the multivariable Cox regression analysis of variables selected from the univariable analysis. We combined conventional PET measures (PMTV) with the proposed distance parameters (either Dmax or Dtotal). The model successfully identified PMTV (HR: 2.57 [1.31, 3.83], *p* = 0.004) and Dmax (HR: 1.98 [1.18, 3.30], *p* = 0.009) as independent predictors for BCR after prostatectomy. The corresponding Concordance Index was 0.68. Examples to interpret the Dmax are displayed in Fig. 4. The figure on the left shows a waterfall plot ranking predicted risk scores of all patients, while the right displays representative cases with low and high Dmax, respectively.

**Table 4:** Multivariable Cox regression models incorporating PET-based features for BCRFS prediction. Three models are compared by the concordance index (C-index)

Models		HR [95% CI]	*P*-value
**Model 0 (C-index 0.65)**			
PSA (> 68.38 ng/ml)	1.01 [0.62, 2.08]		0.070
PMTV (> 41.59 cm^3^)	2.74 [1.62, 4.27]		**0.008**
**Model 1 (C-index 0.68)**			
PSA (> 68.38 ng/ml)	1.22 [0.70, 2.12]		0.170
PMTV (> 41.59 cm^3^)	2.57 [1.31, 3.83]		**0.004**
Dmax (> 9.69 cm)	1.98 [1.18, 3.30]		**0.009**
**Model 2 (C-index 0.68)**			
PSA (> 68.38 ng/ml)	1.49 [0.85, 2.61]		0.170
PMTV (> 41.59 cm^3^)	2.78 [1.58, 4.57]		**0.004**
Dtotal (> 11.95 cm)	1.94 [1.16, 3.24]		**0.011**

## Discussion

Prognostic tools that predict biochemical recurrence are crucial for enhancing treatment management in prostate cancer patients and reducing mortality following primary treatment [22, 23]. Falkenbach et al. [24] demonstrated that the size and SUVmax of nodal lesions are significant contributors to PSA levels in patients with oligorecurrent disease, underscoring the prognostic value of these imaging parameters. Similarly, Zapatero et al. [25] highlighted the challenges of managing nodal oligorecurrence and the pivotal role of advanced imaging techniques, such as PSMA PET/CT, in guiding treatment decisions. On the other hand, patients with heterogeneous PSMA expression might exhibit high PSMA levels even with a low SUVmean at the primary lesion [26]. Recently, the concept of maximum tumor dissemination has gained importance for indicating disease spread in 18 F-FDG PET/CT [27, 28]. It was recognized as the greatest distance between the two most distant hypermetabolic lesions, and has been identified as an independent prognostic factor for survival outcomes [29–32]. In the context of 68Ga-PSMA, studies have investigated the relationship between the distances separating tumor foci and their correlation with MTV and PSA levels [33]. Additionally, distance measures have shown potential in regression analyses for predicting PSA in patients with metastatic castration-resistant prostate cancer (mCRPC) [34]. Nonetheless, there has been no investigation into the prognostic significance of distance metrics for prostate cancer patients. Consistent with previous studies, our retrospective analysis demonstrates the predictive value of high 68Ga-PSMA-11 uptake and tumor volume at the primary lesion. Additionally, the distance from the lesion to the prostate was found to significantly contribute to predicting disease-free survival. We observed correlations between both quantitative and qualitative measures from 68Ga-PSMA-11 PET and pathological parameters. This finding is particularly relevant for patients in whom prostatectomy is not performed, as preoperative PSMA-PET imaging may offer complementary prognostic information when surgical pathology is unavailable.

This study retrospectively collected SUVmax values without standardization, potentially introducing variability in measurements and interpretation. Additionally, the SUVmax values are not directly comparable due to the use of different tracers and imaging equipment across various centers. Despite this, the real-world outcomes are reflected, as the PSMA-PET scans in this study were performed using three different scanner models. Tumor volume and distance to the prostate, however, are less affected by these variations [28]. It is important to note that tumor volume measurements can be influenced by the settings of the delineation tool used. Dmax, as a straightforward feature, is not significantly impacted by lesion contouring, suggesting better reproducibility. Also, unlike sophisticated radiomic features, which are often difficult to interpret from a biological point, Dmax represents the spatial migration of the disease to different anatomical sites.

The combination of Dmax and PMTV enabled the identification of a subgroup with extremely poor prognosis, suggesting that clinicians might need to consider alternative treatment options for these patients. Specifically, patients with both high baseline PMTV (> 41.59 cm^3^) and high Dmax (> 9.69 cm) exhibited significantly worse outcomes, with a 3-year BCRFS of only 6%. This high-risk group accounted for 16.5% of the total events (17/103). Additionally, the combined use of these parameters proved useful for distinguishing low-risk and intermediate-risk patients more effectively than using PMTV measures alone. For the purpose of our analysis, Dmax was categorized into low versus high values during the survival analysis. However, it is important to acknowledge that dichotomizing a continuous variable can result in the loss of valuable information. Further studies are required to validate the relationship between Dmax and the duration of biochemical response.

It should be noted that some patients (5/103) may experience poor BCRFS due to high Gleason scores, even if PET-derived distance features are small. Thus, PSMA-PET should be viewed as a complement to, not a replacement for, pathological assessment. Moreover, while the additional prognostic value of PET-based distance metrics is promising, their integration into routine care must consider cost-effectiveness, particularly in healthcare systems with constrained resources. Future prospective trials are necessary to evaluate whether PET-guided preoperative risk stratification leads to improved clinical outcomes and justifies the cost.

It is also important to acknowledge that PSMA expression can vary substantially between tumor subtypes. Tumors with neuroendocrine differentiation or aggressive dedifferentiated phenotypes may exhibit low PSMA uptake, limiting the sensitivity of PSMA-PET. In such cases, disease extent may be underestimated, and PET-derived features such as SUVmax or Dmax may not reflect the true biological aggressiveness of the tumor. This heterogeneity should be considered in both the interpretation and clinical application of PSMA-PET imaging.

This study has several limitations. Firstly, long-term outcomes (e.g., 5-year BCRFS) were not evaluated, as the median follow-up period was 20 months (ranging from 7 to 36 months). Nevertheless, the study remains significant, as early BCR can serve as a surrogate for prostate cancer-specific mortality [2]. Further studies with extended follow-up are necessary. Secondly, this retrospective analysis is limited to patients from a single center, potentially inducing bias. Nevertheless, the PSMA-PET scans were performed using three different scanner models (Siemens or United Imaging, long-axial field-of-view or regular field-of-view), which supports the robustness of our conclusions. However, caution is still advised when applying our findings to data from PET/MR or other PET/CT systems. Thirdly, the current multivariate model can be integrated with other clinical parameters such as staging information to form a nomogram, which could potentially improve the prediction accuracy but remains to be investigated. Lastly, the study did not consider inter-observer variability in manual delineation, as lesion contours were drawn only once. A comparison of delineations by experienced physicians is recommended to avoid possible bias.

## Conclusion

This study demonstrates the incremental predictive value of the distance from the lesion to the prostate, when forecasting disease-free survival in prostate cancer patients post-primary treatment. The results suggest the potential necessity for alternative treatment strategies for those with abnormal distance features, who are at significantly increased risk of biochemical recurrence.

## Data Availability

No datasets were generated or analysed during the current study.

## Abbreviations

BCR: Biochemical recurrence
BCRFS: BCR-free survival
C-index: Concordance index
CI: Confidence intervals
Dmax: The maximum lesion distance to the prostate
Dtotal: The total lesion distance to the prostate
GS: Gleason score
HR: Hazard ratio
IQR: Interquartile range
mCRPC: Metastatic castration-resistant prostate cancer
MTV: Metabolic tumor volume
OR: Odds ratios
PMTV: Primary metabolic tumor volume
PSA: Prostate-specific antigen
SD: Standard deviation
